# Transcranial Magnetic Stimulation with Intermittent Theta Burst Stimulation Alters Corticospinal Output in Patients with Chronic Incomplete Spinal Cord Injury

**DOI:** 10.3389/fneur.2017.00380

**Published:** 2017-08-04

**Authors:** Hunter J. Fassett, Claudia V. Turco, Jenin El-Sayes, Tea Lulic, Steve Baker, Brian Richardson, Aimee J. Nelson

**Affiliations:** ^1^Department of Kinesiology, McMaster University, Hamilton, ON, Canada; ^2^Division of Physical Medicine and Rehabilitation, Department of Medicine, McMaster University, Hamilton, ON, Canada

**Keywords:** neuroplasticity, transcranial magnetic stimulation, spinal cord injury, placebo, sensorimotor cortex, TBS

## Abstract

Intermittent theta burst stimulation (iTBS) is intended primarily to alter corticospinal excitability, creating an attractive opportunity to alter neural output following incomplete spinal cord injury (SCI). This study is the first to assess the effects of iTBS in SCI. Eight individuals with chronic incomplete SCI were studied. Sham or real iTBS was delivered (to each participant) over primary motor and somatosensory cortices in separate sessions. Motor-evoked potential (MEP) recruitment curves were obtained from the flexor carpi radialis muscle before and after iTBS. Results indicate similar responses for iTBS to both motor and somatosensory cortex and reduced MEPs in 56.25% and increased MEPs in 25% of instances. Sham stimulation exceeded real iTBS effects in the remaining 18.25%. It is our opinion that observing short-term neuroplasticity in corticospinal output in chronic SCI is an important advance and should be tested in future studies as an opportunity to improve function in this population. We emphasize the need to re-consider the importance of the direction of MEP change following a single session of iTBS since the relationship between MEP direction and motor function is unknown and multiple sessions of iTBS may yield very different directional results. Furthermore, we highlight the importance of including sham control in the experimental design. The fundamental point from this pilot research is that a single session of iTBS is often capable of creating short-term change in SCI. Future sham-controlled randomized trials may consider repeat iTBS sessions to promote long-term changes in corticospinal excitability.

## Introduction

Following spinal cord injury (SCI), damage to the ascending and descending spinal pathways leads to sensory and motor impairments below the level of injury. Opportunities to recapture or improve neural output to impaired muscles are of utmost importance, particularly for muscles of the upper limb to allow for greater independence performing activities of daily living. Repetitive transcranial magnetic stimulation (TMS) may provide an opportunity to promote motor recovery in incomplete SCI by strengthening synaptic connectivity within intact descending fibers thereby increasing neural output to affected muscles. To date, repetitive TMS protocols have shown mixed results when assessing recovery of function following SCI (1, 2) with only one report showing increases in corticospinal output (3). Spinal associative plasticity that pairs TMS and nerve stimulation increases neural output to muscles of the hand and improves function (4, 5).

Patterned, rapid delivery of TMS is delivered in a protocol called intermittent theta burst stimulation (iTBS). iTBS delivered over primary motor cortex (M1) facilitates corticospinal output as measured by increases in the amplitude of motor-evoked potentials (MEPs) (6–8) but is also equally effective at reducing MEPs (7). Therefore, caution should be taken when assuming that iTBS should evoke a particular directional effect. Further, there is no clear relationship between the direction of iTBS-induced changes in MEPs and predictable change in motor behavior. However, to date, the evidence suggests that iTBS does indeed alter corticospinal output in healthy, uninjured controls. The advantage of iTBS relative to other TMS approaches is the short duration required to deliver the protocol (~2 min) and its low intensity, making it attractive for both experimenters and participants. To date, no study has examined the effects of iTBS in individuals with incomplete SCI, yet this technique has the potential to alter corticospinal output to impaired muscles of the arm.

We present a sham-controlled pilot study to provide the first characterization of iTBS-induced effects in chronic, incomplete cervical SCI. We consider these pilot data timely since recent SCI research in rodents indicates that repeat sessions of iTBS lead to facilitation of MEPs (9). Before repeat iTBS is delivered in humans with SCI, it is important to demonstrate whether a single session of iTBS is capable of inducing short-term changes as observed in uninjured controls (7, 10–12). To test this, we delivered real and sham iTBS over M1, and real iTBS was delivered over primary somatosensory cortex (S1). We included S1 as a novel target since iTBS targeting S1 has primarily shown facilitation of sensory physiology (13–15) and improvement of sensory discrimination (15, 16). Given that neuroplasticity involving reorganization in SCI likely occurs in both M1 and S1, we considered that stimulation over either area may influence the excitability of adjacent M1 to ultimately alter the neural output to the target muscles.

## Methods

Eight individuals with chronic (>1 year post-injury) incomplete cervical (injury from C4–T1, ASIA classification as B, C, or D) SCI participated (Table 1) in three experimental sessions. Subjects were screened for contraindications to TMS prior to participation. The study conformed to the declaration of Helsinki and was approved by the Hamilton Integrated Research Ethics Board. All individuals provided written consent prior to participation.

**Table 1:** Participant demographics and results.

ID	Age	Gender	Injury level	Years post-injury	ASIA	Medications	iTBS site	MEP
1	29	M	C4	5	C	Baclofen	M1 S1	↓ (21.8%) Sham > real
2	39	M	C6–C7	14	C	Baclofen	M1 S1	↑ (4.0%) ↓ (20.3%)
3	26	M	C5	4.5	C	Fesoterodine	M1 S1	↓ (30.8%) ↓ (26.7%)
4	41	M	C6	2	D	Diazepam, pregabalin, cyclobenzaprine	M1 S1	Sham > real ↓ (15.9%)
5	39	M	C5	39	N/A	None	M1 S1	↑ (73.9%) ↓ (25.3%)
6	55	F	C3–C4	2	C	Gabapentin, citalopram	M1 S1	Sham > real ↑ (20.9%)
7	58	M	C6–C7	33	B	Baclofen, clonazepam	M1 S1	↓ (55.0%) ↑ (13.1%)
8	68	M	C4	3	B	Baclofen, pregabalin	M1 S1	↓ (3.9%) ↓ (21.4%)
						**Percent of instances demonstrating change (*N* = 16)**		**MEP**
						↑		25%
						↓		56.25%
						Sham > Real		18.75%

*Participant demographic information and individual responses to iTBS are shown*.

*M, male; F, female; C, cervical spine (i.e., C4); iTBS, intermittent theta burst stimulation; M1, primary motor cortex; S1, primary somatosensory cortex; ASIA, American Spinal Injury Association Impairment Scale (A = no sensory or motor function preserved in sacral segments; B = sensory function is preserved with no motor function; C = sensory function is preserved below the level of injury, most muscles below injury have a grade less than 3; D = motor function is preserved below the level of injury, most muscles below injury have a grade of 3 or more; E = normal sensory and motor function); RMT, resting motor threshold; MEP, motor-evoked potential; ↑, measure is increased following iTBS relative to sham effect (percentage of change exceeding sham effects); ↓, measure is decreased following iTBS relative to sham effect (percentage of change exceeding sham effects); N/A, data could not be obtained*.

**Figure 1:**
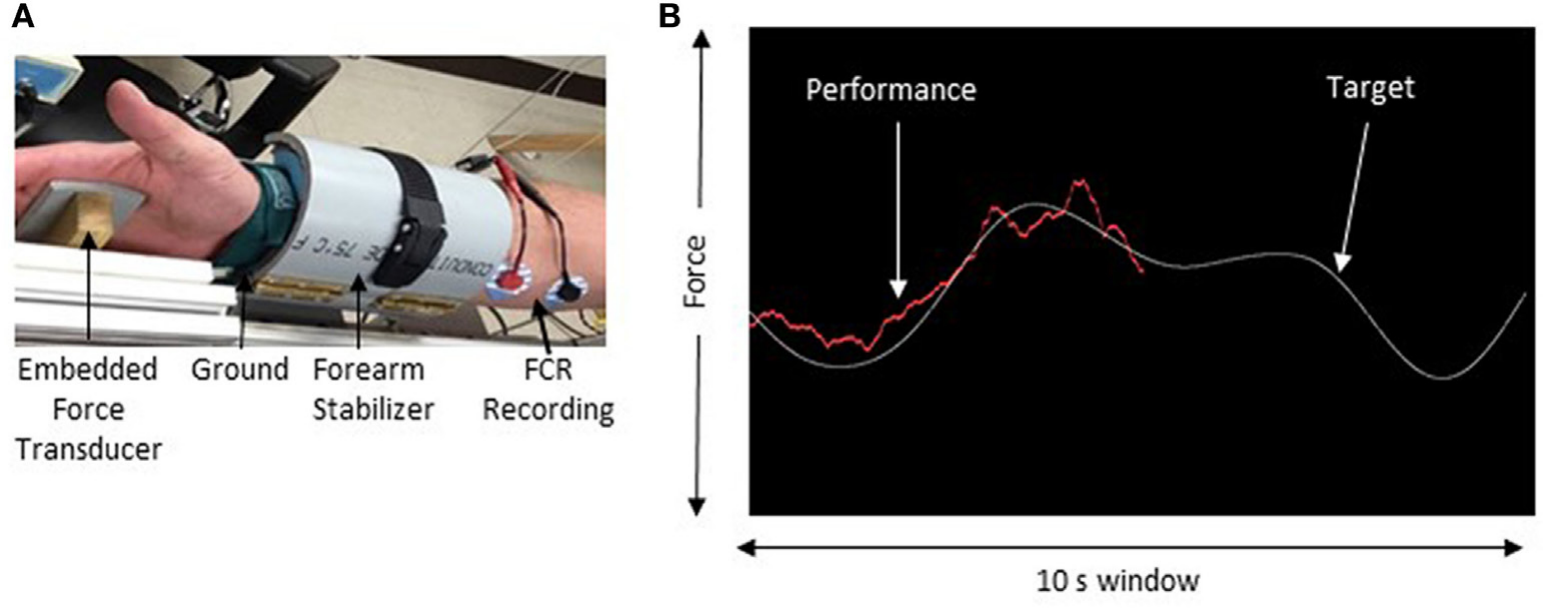
Experimental setup. **(A)** The apparatus used to maintain forearm position during force tracking. **(B)** Sample of the force tracking task. The white line represents the target waveform and the red line is controlled by the force transducer and represents the participant’s performance.

Electromyography (EMG) was recorded using surface electrodes (9 mm diameter Ag–AgCl) that were placed in a belly–belly montage on the flexor carpi radialis (FCR) and extensor carpi radialis muscles with a wet ground placed around the forearm proximal to the recording electrodes (Figure 1). All EMG recordings were band-pass filtered between 20 Hz and 2.5 kHz, amplified 1,000× (Intronix Technologies Corporation Model 2024F, Bolton, Canada), and digitized using an analog-to-digital interface at 5 kHz (Power1401, Cambridge Electronics Design, Cambridge, UK). Data were collected using Signal software (v6.02, Cambridge Electronics Design, Cambridge, UK).

In this study, iTBS was delivered over the hemisphere contralateral to the least impaired FCR muscle. To determine the least impaired FCR, maximum voluntary contraction was recorded in three trials from the FCR muscle while maintaining a maximum isometric contraction against an immovable post. Each trial consisted of MVC for 5 s followed by 1 min of rest. Visual feedback from an oscilloscope (Tektronix TDS2004c, USA) displayed muscle activity to the participant. The maximum peak-to-peak amplitude achieved across all trials was documented as the MVC. The FCR that produced the greatest MVC was taken as the least impaired limb.

Single-pulse TMS was delivered *via* a 50 mm diameter figure-of-eight branding coil connected to a Magstim 200^2^ stimulator (Magstim, UK) over the optimal location (i.e., motor hotspot) to elicit MEPs in the relaxed FCR of the least affected arm. The coil was positioned 45° in relation to the parasagittal plane to induce posterior-to-anterior current in the cortex. The motor hotspot was marked by digital registration using a standard MRI template *via* Brainsight Neuronavigation (Rogue Research, Canada). At this location, resting motor threshold (RMT) was quantified as the percentage of maximum stimulator output that elicited MEPs ≥50 µV peak-to-peak amplitude in 5 out of 10 consecutive trials (17). Active motor threshold (AMT) was determined as the percentage of maximum stimulator output that produced an MEP of ≥200 µV peak-to-peak amplitude in 5 out of 10 consecutive trials while participants maintained a contraction of 15% of their MVC.

Intermittent theta burst stimulation protocol was delivered using a 70 mm inner diameter figure-of-eight coil with a Magstim Super Rapid^2^ Plus (Magstim, Whitland, UK) using biphasic pulses in bursts of three pulses delivered at 30 Hz, in 6 Hz trains that lasted 2 s, that was followed by a period of 8 s in which no pulses were delivered (8). iTBS was repeated for a total of 612 pulses delivered at 80% AMT. Participants received one of three iTBS interventions in each session and the order of delivery was pseudorandomized across participants: iTBS-M1 delivered at the motor hotspot for the least affected FCR, iTBS-S1 delivered at a position digitally marked 2 cm posterior to the FCR motor hotspot, and sham iTBS delivered over the FCR motor hotspot. This sham coil appears and sounds like verum iTBS but does not deliver real pulses. All TBS (including real and sham) was delivered to the hemisphere contralateral to the target FCR muscle (i.e., the least affected).

Motor-evoked potential recruitment curves were recorded before and immediately following the iTBS intervention in each session. Single-pulse TMS was applied over the FCR motor hotspot at 8 different stimulus intensities: 90, 100, 110, 120, 130, 140, 150, and 160% of RMT. Three pulses were delivered at each intensity in a randomized order. The average area of the MEP was quantified at each intensity by identifying the area under the MEP in each trial within a 30 ms window. The area of the MEP was measured.

To assess the effects of iTBS across the entire recruitment curve, we quantified the area under the MEP recruitment curve (AURC) for each participant as this has been shown to be a reliable way to assess corticospinal excitability changes in proximal upper arm muscles (18). For MEP recruitment curves, we calculated the percent change from T0 to T1 following real iTBS to either M1 or S1 and subtracted the percent change from T0 to T1 obtained from the sham condition. Therefore, on a case-by-case basis, we only considered an effect to be “real” if it exceeded the effect of sham, or if it was in the direction opposite to sham effects (Eq. 1).

(1) $$\text{\% Change}=100\times \left(\frac{\text{T}1_{\text{real}}}{\text{T}0_{\text{real}}}-1\right)-100\times \left(\frac{\text{T}1_{\text{sham}}}{\text{T}0_{\text{sham}}}-1\right)$$

## Results

The effects of real and sham iTBS are shown in Table 1 for each participant. We observed no differences associated with the location of stimulation (S1 versus M1); MEPs decreased in 50 and 62.5% of subjects for M1 and S1, respectively, and increased in 25% of participants for both M1 and S1. In total, following subtraction of sham effects, iTBS decreased MEPs in 56.25% of instances (9/16) and increased MEPs in 25% of instances. Real iTBS did not exceed the effects of sham in the remaining 18.75% (Table 1).

## Discussion

The data demonstrate that iTBS over M1 and/or S1 led to changes in MEPs in 81.25% of instances, and these were changes that exceeded those induced by sham stimulation. The present data from eight individuals with chronic SCI indicate that a single session of iTBS over M1 or the adjacent SI tends to decrease MEPs in FCR. These data are promising since they indicate that corticospinal excitability is modifiable in this population. We note that sham effects exceeded real iTBS effects in ~20% of instances and this finding highlights the importance of including sham control stimulation in future studies. One limitation is the lack of imaging data to quantify the spinal cord lesion characteristics in our participants.

Previous research suggests that iTBS should be expected to increase MEPs (7). However, in a larger sample study, iTBS was shown to suppress MEPs in 48% and increase in 52%, leading to nearly proportional outcomes (10). Further, it is unclear whether increases or decreases in MEPs have direct relationships with motor performance from a given muscle. Therefore, in the present study, our focus was not on the direction of change induced by iTBS but rather whether MEPs were indeed modifiable with iTBS in chronic SCI. Our data from our sample of individuals indicate that corticospinal excitability to an impaired muscle in SCI is modifiable, and this information is important since iTBS is of minimal imposition to a participant requiring low-intensity stimulation for less than 3 min. Work in other clinical populations has shown that iTBS may not be as effective. Notably, iTBS does not alter MEPs in Tourette’s syndrome (19, 20), focal hand dystonia (21), multiple system atrophy (22), Alzheimer’s disease (23), and Parkinson’s disease (24). However, another study demonstrated suppression of MEPs in individuals with Parkinson’s diseases when iTBS is delivered to the more affected hemisphere (25). The data from this pilot study indicate that iTBS may provide an opportunity for inducing changes in corticospinal excitability, in line with recent iTBS study in rodent model (9), albeit we did not typically see facilitation. This may relate to the single versus multiple trains of iTBS between the studies, or differences across species.

We conclude that a single session of iTBS tends to decrease MEPs in individuals with incomplete chronic SCI. Our findings highlight the need to reevaluate our expectation of the effects of a single session of iTBS effects in SCI as we observed suppression more frequently than facilitation following a single bout of stimulation. It is our opinion that these pilot data are promising since they suggest that changes in corticospinal excitability are possible in chronic SCI, which creates an avenue for future research in this population. These data may assist with the development and design of future, larger-scale studies regarding the anticipated effects of iTBS in chronic SCI.

## Ethics Statement

The study conformed to the declaration of Helsinki and was approved by the Hamilton Integrated Research Ethics Board.

## Author Contributions

HF conceived of the study, collected, analyzed, and interpreted data, and drafted the manuscript. CT and JE-S collected, analyzed, and interpreted data and edited manuscript. TL collected data and edited manuscript. BR designed technical aspects and edited manuscript. SB provided oversight on SCI participants and edited manuscript. AN conceived of the study, analyzed and interpreted data, and drafted the manuscript.

## Conflict of Interest Statement

The authors declare that the research was conducted in the absence of any commercial or financial relationships that could be construed as a potential conflict of interest.
